# Characteristics associated with dietary patterns in Brazilian children under two years of age

**DOI:** 10.11606/s1518-8787.2022056003757

**Published:** 2022-11-18

**Authors:** Rumão Batista Nunes de Carvalho, Maria Laura da Costa Louzada, Fernanda Rauber, Renata Bertazzi Levy

**Affiliations:** I Universidade Federal do Piauí Faculdade de Enfermagem Picos PI Brasil Universidade Federal do Piauí. Faculdade de Enfermagem. Picos, PI, Brasil; II Universidade de São Paulo Faculdade de Saúde Pública Núcleo de Pesquisas Epidemiológicas em Nutrição e Saúde São Paulo SP Brasil Universidade de São Paulo. Faculdade de Saúde Pública. Núcleo de Pesquisas Epidemiológicas em Nutrição e Saúde. São Paulo, SP, Brasil; III Universidade de São Paulo Faculdade de Medicina Departamento de Medicina Preventiva São Paulo SP Brasil Universidade de São Paulo. Faculdade de Medicina. Departamento de Medicina Preventiva. São Paulo, SP, Brasil

**Keywords:** Infant Nutrition, Diet, Food, and Nutrition, Socioeconomic Factors, Child Health Services

## Abstract

**OBJECTIVE:**

To analyze the dietary patterns of Brazilian children under two years of age and assess their association with sociodemographic characteristics and health service use.

**METHODS:**

This is a cross-sectional study with data from the 2013 National Health Survey (PNS). Patterns were found for two age groups by principal component analysis and their correlation with characteristics of interest was tested by linear regression models.

**RESULTS:**

We found two dietary patterns for our groups. The first consisted of the consumption of fresh or minimally processed foods and the second, of ultra-processed foods. The greater adherence of children between six and 11 months to the first pattern was associated with higher *per capita* family income and urban residences in the most developed regions of Brazil. At 12 months or more, adherence related to white race/color, higher *per capita* family incomes, residence in more developed regions, and visits to private childcare. Adherence to the second pattern among children under one year of age was inversely associated with Yellow or Indigenous race/color, residence in the Brazilian Northeast, and childcare in specialized public or private services. At 12 months or more, greater adherence was directly associated with Black or Brown children who resided in more developed regions, and inversely associated with those living in the Brazilian Northeast.

**CONCLUSION:**

We found two opposite dietary patterns in Brazilian children under two years of age and that several social determinants modify their chance of adhering to these patterns.

## INTRODUCTION

Children’s first two years of life are crucial for their growth and development. At this stage, adequate and healthy eating plays a fundamental role for the satisfactory occurrence of these processes^1^. As long as it is offered in appropriate quantities and quality, food is associated with the best health conditions in childhood and can prevent several diseases which develop in adulthood^1^.

On the other hand, inadequate nutrition is the most important risk factor for the loss of life years and is associated with several highly prevalent chronic non-communicable diseases, such as obesity, type 2 diabetes, hypertension, and some types of cancer^2^. Moreover, it is one of the main determinants of the global syndemic of obesity, malnutrition, and climate change, affecting most people in the world^3^. These consequences are particularly relevant for children, since health behaviors and damages acquired in the early stages of life tend to have repercussions at later ages^4^.

Previous studies show that substituting the consumption of healthy meals (based on fresh or minimally processed foods) by ready-to-eat or semi-ready ones (i.e., ultra-processed) constitute one of the main determinants of worsening food quality^5,6^. Children’s consumption of unhealthy foods has not only grown but seems also to be associated with a higher risk of developing obesity^7^ and dyslipidemia^8^; the former is the most common metabolic factor in the development of other chronic diseases among children, such as diabetes and high blood pressure^4,9^.

These findings are especially important since children’s first years of life include not only their introduction to complementary foods which are essential to meet their nutritional and developmental needs but also the possibility of establishing parental practices, preferences, and eating habits (whether healthy or not^10^), which also requires the continuous evaluation of consumption markers in this age group.

Moreover, only few studies have evaluated the influence of health service use on infant feeding. In general, studies which evaluated mothers who used this support have focused on identifying aspects related to the monitoring and performance of indicators, especially of breastfeeding^11,12^, and the existence of a link between users and healthcare providers^13^. Moreover, they claim that introducing unrecommended foods in children’s first year of life is associated with healthcare providers failing to effectively guide parents and guardians on nutrition and the latter’s low adherence to implemented nutritional prevention and education actions, especially in primary care services^14^. This scenario also requires further investigations.

Considering that the analysis of the simultaneous effect of different food groups^15^tends to better represent food intake, previous studies conducted in schoolchildren or residents of certain Brazilian regions evaluated food intake by assessing dietary patterns, observing that maternal characteristics tend to influence children’s adherence to these patterns^16,17^. However, these findings neither enable the generalization of the dietary patterns and sociodemographic factors associated with the entire national territory and all age groups in the infant population nor provide evidence of this population’s dietary patterns which may be associated with health service use. This gap enables us to conduct new studies which analyze information in a nationally representative sample of Brazilian children, such as those under two years of age.

Thus, based on population sample data related to infant feeding, made available by the National Survey of Health in 2013^18^, and robust exploratory analysis methods (which would enable us to assess and explain the most frequent food consumption combinations), this study aims to analyze the dietary patterns of Brazilian children under two years of age and evaluate their association with sociodemographic characteristics and health service use.

## METHODS

### Design, Study Population, and Data Collection

This is a cross-sectional, household-based study with secondary data from the Brazilian National Health Survey (PNS), conducted by the *Instituto Brasileiro de Geografia e Estatística* (IBGE – Brazilian Institute of Geography and Statistics), in partnership with the Ministry of Health. Data were collected by trained interviewers from August 2013 to February 2014.

PNS used a three-stage probabilistic sampling: first, primary sampling units were composed of census tracts; then, households were drawn; and finally, residents aged 18 years or older. At each stage, selection was conducted by simple random sampling. PNS census tracts or sets were drawn via a master sample based on IBGE Integrated System of Household Surveys, with greater geographic spread and estimate precision. Thus, PNS represents Brazil, its macro-regions, federation units, metropolitan regions, and capitals.

Interview records were obtained from 60,202 households (an 86.1% response rate). Children under two years of age were identified in the PNS second stage, in which a fixed number of permanent private households was chosen from each primary sampling unit^19^. Mothers or guardians answered the questions regarding the children in the chosen households. Only 3,646 children between six and 23 months of age were included in the final sample of this study.

Sampling weights were defined for the primary sampling units, households, and all their residents. Additional details about the sampling process, weighting factors, collection, and other information can be found in a previous publication^19^.

### Study Variables

Food intake, sociodemographic characteristics, and health service use data were analyzed.

### Food Intake

Food intake was assessed by a questionnaire, applied to children’s guardians, aiming to evaluate the consumption of the following foods in the 24 hours prior to data collection: non-breast milk or dairy products; fruits or natural juices; vegetables; beans or other legumes (lentils, peas, etc.); meat or eggs; potato and other tubers and roots (sweet potato, cassava); cereals and derivatives (rice, bread, cereal, pasta, flour, etc.); artificial juices; cookies or cake; sweets or other foods with sugar; and soft drinks. All these variables were categorized into dichotomous indicators (0 = did not consume; 1 = consumed).

### Sociodemographic Characteristics

The following sociodemographic variables were considered: sex (male; female); age (6 to 11 months and 29 days; 12 to 23 months and 29 days); race/color (white; Black or Brown; other – Yellow or Indigenous); *per capita* family income (distributed in quintiles); area of residence (rural; urban); and major regions (North; Northeast; Southeast; South; Midwest).

### Health Service Use Characteristics

The following health service use variables were included: health insurance possession (yes; no) and place of children’s growth and development monitoring (childcare) (no follow up; basic health unit; specialized public service - specialty center, public polyclinic or health center, public hospital/outpatient clinic; private clinic; and others).

### Data Analysis

Sociodemographic distribution, health service use, and the prevalence of the food consumed in our sample were shown as percentages and their respective 95% confidence intervals (95%CI).

Food intake patterns were defined via factor analysis by principal components^15^, a method indicated when data show abnormal distribution^20^. Then, the obtained factors were rotated by orthogonal varimax rotation. The Bartlett and Kayser-Meyer-Olkin’s (KMO) tests of sphericity were applied to assess data adequacy for factor analysis^21^. The factors which met an eigenvalue > 1 and scree plot graphical analysis criteria were maintained. Best factor interpretability was used to compose final components and foods with factor loadings equal to or greater than 0.3 were considered important for finding dietary patterns. Foods with cross-loadings – equal to or greater than the specified load value (0.3) in two or more factors – were removed from analysis to identify and isolate data which best fit our factorial structure^20^. Then, factorial scores were predicted for each evaluated child.

Associations between predicted scores (dependent variable), sociodemographic variables, and health service use (independent) were evaluated by linear regression models. In our adjusted model, variables with p < 0.20 were included in bivariate analyses, in which only the significant analyses were maintained (p < 0.05). Model adjustment was assessed by residue distribution analysis, which showed normality.

Data were analyzed in Stata, version 14.0, via its survey module, considering our sample design. Factor analysis and linear regression were stratified by age in months and performed for our whole sample.

PNS was approved by the National Research Ethics Commission (Protocol 328,159, of June 26, 2013). All interviewees were contacted and informed of the research and those who agreed to participate in this research signed informed consent forms.

## RESULTS

We evaluated 3,646 Brazilian children between six and 23 months (1,249 from six to 11 months; 2,397 from 12 to 23 months). Most were Black or Brown (51.9%) boys (53.3%) aged between 12 and 23 months (68.3%) and belonging to the first and second quintiles of our *per capita* family income distribution (52.6%). Children living in urban areas (83.8%) in the Brazilian Northeast (29.4%) and Southeast (37.2%) without health insurances (73.6%) and who had visited basic health units (55.4%) and private services (27.9%) showed the highest distribution frequencies (Table 1).

**Table 1:** Characteristics of children aged six months to less than two years of age. National Survey on Health, 2013, (n = 3,646).

Characteristics	Percentage (%)	95%CI
**Sociodemographic**		
Gender		
Male	53.3	50.7–55.8
Women	46.7	44.2–49.3
Age		
6–11 months and 29 days	31.7	29.3–34.1
12–23 months and 29 days	68.3	65.9–70.7
Race/color		
White	47.1	44.6–49.6
Black or Brown	51.9	49.3–54.4
Other^a^	1.0	0.5–1.9
*Per capita* family income		
1st quintile (lowest income)	26.9	25.0–29.0
2nd quintile	25.7	23.5–28.0
3rd quintile	21.5	19.5–23.6
4th quintile	13.5	11.8–15.5
5th quintile (highest income)	12.4	10.5–14.4
Area of residence		
Rural	16.2	14.8–17.6
Urban	83.8	82.4–85.2
Region of the Country		
North	11.0	10.3–11.8
Northeast	29.4	27.9–31.0
Southeast	37.2	35.5–38.9
Sul	15.6	14.4–16.9
Midwest	6.8	6.2–7.3
**Health service use**		
Health insurance coverage		
Yes	26.4	24.0–29.0
No	73.6	71.0–76.0
Childcare		
No follow up	7.3	6.1–8.8
Basic health unit	55.4	52.6–58.0
Specialized public service^b^	6.7	5.5–8.1
Private office or clinic	27.9	25.4–30.5
Other	2.7	2.0–3.7

^a^ Yellow or Indigenous

^b^ Specialty center, public polyclinic or health center; public hospital/outpatient clinic.

Table 2 describes these children’s food consumption. We observed that the highest percentages of consumed fresh or minimally processed food consisted of cereals and derivatives (83.1%), fruits or natural fruit juices (81.5%), and non-maternal milk and its derivatives (80.5%). The most consumed ultra-processed foods were cookies/cake (76.6%) and more than half of children under two years of age (52.4%) had consumed artificial juices and soft drinks within 24 hours before data collection.

**Table 2:** Percentage of consumption in the 24 hours prior to data collection in our sample with children aged six months to less than two years of age. National Survey on Health, 2013, (n = 3,646).

Consumed food	Percentage (%)	95%CI
Cereals and derivatives (rice, bread, cereal, pasta, flour, etc.)	83.1	81.1–84.9
Fruit or fruit juice	81.5	79.5–83.3
Non-breast milk or derivatives	80.5	78.3–82.5
Beans or other legumes (lentils, peas, etc.)	79.3	77.2–81.2
Biscuits or cake	76.6	74.3–78.7
Meat or eggs	76.1	73.9–78.2
Leaf vegetables	74.8	72.6–76.9
Potato and other tubers and roots (sweet potato, cassava)	61.1	58.6–63.5
Sweets, candies or other sugary foods	39.5	36.9–42.1
Artificial juices	31.7	29.4–34.1
Soft drinks	20.7	18.7–22.9

Our data adequacy assessment showed a 0.80 KMO value and Bartlett results with p < 0.001, indicating adequacy for factor analysis.

Our analysis showed two factors, both in our total sample and in the stratified one according to age. Both factors explained 46% of the shared variance in children between six and 11 months and 41% in older ones. Table 3 shows the rotated factor loadings in each of the two components for the total sample and the stratified one. Consumption of “cookies or cake” showed cross-loading. Thus, we removed it from our analysis to better distinguish the validity between factors.

**Table 3:** Factorial loads rotated for the two components in our principal component analysis stratified by age. National Health Survey (PNS), 2013.

	6–11 months and 29 days (n = 1,249)			12–23 months and 29 days (n = 2,397)			Total sample (n = 3,646)		
		
	Minimally processed pattern	Ultra-processed pattern	Common percentage^a^	Minimally processed pattern	Ultra-processed pattern	Common percentage	Minimally processed pattern	Ultra-processed pattern	Common percentage
**Consumed food**									
Non-breast milk or derivatives	**0.35**	-0.07	0.87	**0.38**	-0.02	0.86	**0.36**	0.04	0.87
Fruit or fruit juice	**0.64**	-0.05	0.59	**0.57**	-0.09	0.67	**0.60**	-0.06	0.63
Leaf vegetables	**0.75**	-0.07	0.44	**0.71**	-0.06	0.49	**0.73**	-0.08	0.46
Beans or other legumes	**0.61**	0.23	0.58	**0.60**	0.16	0.61	**0.60**	0.23	0.59
Meat or eggs	**0.69**	0.21	0.47	**0.65**	0.22	0.53	**0.65**	0.28	0.50
Potato and other tubers and roots	**0.75**	0.00	0.44	**0.63**	0.11	0.60	**0.67**	0.02	0.55
Cereals and derivatives	**0.61**	0.25	0.56	**0.60**	0.26	0.57	**0.60**	0.29	0.56
Artificial juices	0.03	**0.69**	0.53	0.06	**0.64**	0.59	0.04	**0.67**	0.56
Sweets, candies or other sugary foods	0.18	**0.70**	0.48	0.13	**0.73**	0.45	0.14	**0.74**	0.44
Soft drinks	-0.02	**0.75**	0.44	0.02	**0.73**	0.46	0.00	**0.73**	0.46
**Proportional variance (%)**	29.00	17.00	-	25.00	16.00	-	26.00	18.00	-
**Accumulated variance (%)**	29.00	46.00	-	25.00	41.00	-	26.00	44.00	-

^a^ Commonality: proportion of the variance of each variable explained by the extracted factors.

The items indicated in bold showed a factorial load equal to or greater than 0.3.

In both age groups and the total sample, the first component – “minimally processed pattern” – included the consumption of fresh or minimally processed foods (non-maternal milk or derivatives, fruits or natural juices, vegetables, beans or other legumes, meat or eggs, potatoes and other tubers and roots, and cereals and derivatives). The second one, “ultra-processed pattern,” included ultra-processed foods (artificial juices, sweets, candies or other sugary foods and soft drinks).

Our crude models showed that adherence to the minimally processed pattern in both age groups was higher among urban white children in higher-income families who resided in the more developed regions of Brazil, had health insurances, and had visited in private health services. Our adjusted models for children aged six to 11 months maintained this pattern, which showed significant associations with higher-income families who resided in urban areas in the more developed Brazilian regions. The model for children aged 12 to 24 months showed that their greater adherence to the minimally processed pattern was significantly associated with white children from higher-income families who resided in more developed regions and visited private health services (Tables 4 and 5).

**Table 4:** Crude and adjusted association between food consumption patterns in children aged six to 11 months and 29 days, sociodemographic characteristics, and access to health services. National Survey on Health, 2013, (n = 1,249).

Características	Minimally processed pattern				Ultra-processed pattern			
	
	Crude β^c^ (95%CI)	p	Adjusted β^d^ (95%CI)	p	Crude β^c^ (95%CI)	p	Adjusted β^d^ (95%CI)	p
**Sociodemographic**								
*Gender*								
Male	Ref.		Ref.		Ref.		Ref.	
Women	-0.08 (-0.25 to 0.09)	0.361	-		-0.03 (-0.19 to 0.13)	0.718	-	-
*Race/color*								
White	Ref.		Ref.		Ref.		Ref.	
Black or Brown	-0.28 (-0.42 to -0.13)	< 0.001	-0.00 (-0.16 to 0.15)	0.971	0.12 (-0.05 to 0.29)	0.152	0.11 (-0.06 to 0.29)	0.190
Other^a^	-0.07 (-0.68 to 0.54)	0.821	-0.15 (-0.81 to 0.52)	0.666	-0.52 (-0.89 to -0.14)	0.007	-0.40 (-0.77 to -0.02)	0.039
*Per capita household income*								
1st quintile (lowest income)	Ref.		Ref.		Ref.		Ref.	
2nd quintile	0.51 (0.29 to 0.74)	0.000^e^	0.31 (0.10 to 0.53)	< 0.001^e^	0.06 (-0.15 to 0.28)	< 0.001^e^	0.07 (-0.15 to 0.29)	0.060^e^
3rd quintile	0.76 (0.56 to 0.96)		0.44 (0.21 to 0.68)		-0.02 (-0.30 to 0.26)		0.01 (-0.30 to 0.32)	
4th quintile	0.83 (0.63 to 1.03)		0.43 (0.18 to 0.68)		-0.17 (-0.37 to 0.03)		-0.13 (-0.38 to 0.11)	
5th quintile (highest income)	1.03 (0.83 to 1.23)		0.53 (0.27 to 0.78)		-0.49 (-0.66 to -0.33)		-0.31 (-0.59 to -0.03)	
*Area of residence*								
Rural	Ref.		Ref.		Ref.		Ref.	
Urban	0.51 (0.29 to 0.74)	< 0.001	0.40 (0.21 to 0.60)	< 0.001	-0.11 (-0.34 to 0.11)	0.322	-	-
*Region of the Country*								
North	Ref.		Ref.		Ref.		Ref.	
Northeast	0.33 (0.12 to 0.55)	0.003	0.35 (0.14 to 0.55)	0.001	-0.26 (-0.46 to -0.05)	0.013	-0.25 (-0.43 to -0.06)	0.010
Southeast	0.80 (0.58 to 1.03)	< 0.001	0.54 (0.31 to 0.78)	< 0.001	-0.08 (-0.34 to 0.18)	0.528	0.11 (-0.16 to 0.38)	0.421
South	0.81 (0.60 to 1.01)	< 0.001	0.54 (0.33 to 0.74)	< 0.001	0.02 (-0.25 to 0.29)	0.895	0.22 (-0.04 to 0.47)	0.096
Midwest	0.83 (0.61 to 1.05)	< 0.001	0.60 (0.38 to 0.81)	< 0.001	0.06 (-0.22 to 0.35)	0.671	0.20 (-0.07 to 0.78)	0.146
**Health service use**								
*Health insurance coverage*								
Yes	Ref.		Ref.		Ref.		Ref.	
No	-0.54 (-0.69 to -0.39)	< 0.001	-0.11 (-0.28 to 0.06)	0.211	0.23 (0.07 to 0.40)	0.006	-0.02 (-0.22 to 0.18)	0.842
*Childcare*								
No follow up	Ref.		Ref.		Ref.		Ref.	
Basic health unit	0.45 (0.13 to 0.76)	0.005	0.15 (-0.16 to 0.46)	0.345	-0.17 (-0.47 to 0.12)	0.252	-0.17 (-0.47 to 0.13)	0.272
Specialized public service^b^	0.29 (-0.12 to 0.71)	0.167	0.04 (-0.33 to 0.42)	0.820	-0.41 (-0.78 to -0.03)	0.032	-0.40 (-0.78 to -0.01)	0.043
Private office or clinic	0.96 (0.65 to 1.30)	< 0.001	0.30 (-0.04 to 0.65)	0.086	-0.65 (-0.94 to -0.36)	< 0.001	-0.57 (-0.93 to -0.21)	0.002
Other	0.09 (-0.39 to 0.57)	0.710	-0.04 (-0.44 to 0.37)	0.864	-0.33 (-0.80 to 0.14)	0.167	-0.32 (-0.77 to 0.13)	0.159

Note: Significant values: p < 0.05.

^a^ Yellow or Indigenous.

^b^ Specialty center, public polyclinic or health center; public hospital/outpatient clinic.

^c^ Crude regression coefficient.

^d^ Regression coefficient adjusted for sociodemographic variables and health service use with p < 0.20 in bivariate analysis.

^e^ Test for linear trend.

**Table 5:** Crude and adjusted association between food consumption patterns in children aged 12–23 months and 29 days and sociodemographic characteristics and health services accessibility. National Survey on Health, 2013, (n = 2,397).

Characteristics	Minimally processed pattern				Ultra-processed pattern			
	
	Crude β^c^ (95%CI)	p	Adjusted β^d^ (95%CI)	p	Crude β^c^ (95%CI)	p	Adjusted β^d^ (95%CI)	p
**Sociodemographic**								
*Gender*								
Male	Ref.		Ref.		Ref.		Ref.	
Women	0.07 (-0.04 to 0.18)	0.231	-	-	0.06 (-0.06 to 0.19)	0.312	-	-
*Race/color*								
White	Ref.		Ref.		Ref.		Ref.	
Black or Brown	-0.37 (-0.48 to -0.26)	< 0.001	-0.13 (-0.24 to -0.01)	0.030	0.14 (0.03 to 0.26)	0.015	0.17 (0.05 to 0.29)	0.005
Other^a^	-0.30 (-0.78 to 0.17)	0.213	-0.09 (-0.52 to 0.33)	0.670	0.35 (-0.09 to 0.79)	0.120	0.26 (-0.14 to 0.66)	0.204
*Per capita household income*								
1st quintile (lowest income)	Ref.		Ref.		Ref.		Ref.	
2nd quintile	0.33 (0.18 to 0.48)	< 0.001^e^	0.23 (0.07 to 0.39)	< 0.001^e^	0.04 (-0.12 to 0.20)	0.012^e^	-0.04 (-0.20 to 0.13)	0.068^e^
3rd quintile	0.60 (0.44 to 0.76)		0.38 (0.21 to 0.55)		0.05 (-0.14 to 0.24)		-0.01 (-0.20 to 0.18)	
4th quintile	0.72 (0.56 to 0.89)		0.45 (0.27 to 0.63)		0.07 (-0.12 to 0.27)		0.03 (-0.19 to 0.25)	
5th quintile (highest income)	0.90 (0.74 to 1.06)		0.54 (0.34 to 0.74)		-0.37 (-0.57 to -0.18)		-0.38 (-0.64 to -0.13)	
*Area of residence*								
Rural	Ref.		Ref.		Ref.		Ref.	
Urban	0.39 (0.25 to 0.53)	< 0.001	0.13 (-0.02 to 0.27)	0.082	0.03 (-0.10 to 0.16)	0.648	-	-
*Region of the Country*								
North	Ref.		Ref.		Ref.		Ref.	
Northeast	0.30 (0.11 to 0.49)	0.002	0.30 (0.12 to 0.48)	0.001	-0.17 (-0.32 to -0.02)	0.022	-0.16 (-0.31 to -0.02)	0.027
Southeast	0.70 (0.50 to 0.89)	< 0.001	0.43 (0.24 to 0.63)	< 0.001	0.08 (-0.09 to 0.26)	0.339	0.25 (0.07 to 0.43)	0.006
South	0.77 (0.58 to 0.96)	< 0.001	0.54 (0.35 to 0.73)	< 0.001	0.24 (0.05 to 0.44)	0.016	0.41 (0.20 to 0.62)	< 0.001
Midwest	0.72 (0.52 to 0.93)	< 0.001	0.53 (0.33 to 0.73)	< 0.001	0.12 (-0.08 to 0.32)	0.254	0.23 (0.03 to 0.43)	0.026
**Health service use**								
*Health insurance coverage*								
Yes	Ref.		Ref.		Ref.		Ref.	
No	-0.47 (-0.58 to -0.36)	< 0.001	-0.06 (-0.20 to 0.08)	0.395	0.14 (-0.00 to 0.29)	0.053	0.03 (-0.15 to 0.21)	0.734
*Childcare*								
No follow up	Ref.		Ref.		Ref.		Ref.	
Basic health unit	0.27 (0.06 to 0.48)	0.011	0.16 (-0.03 to 0.35)	0.093	-0.03 (-0.23 to 0.16)	0.738	-0.05 (-0.25 to 0.15)	0.614
Specialized public service^b^	0.29 (0.00 to 0.58)	0.047	0.16 (-0.12 to 0.43)	0.261	0.00 (-0.26 to 0.26)	0.985	-0.01 (-0.27 to 0.24)	0.922
Private office or clinic	0.76 (0.55 to 0.97)	< 0.001	0.27 (0.05 to 0.49)	0.015	-0.23 (-0.44 to -0.01)	0.043	-0.19 (-0.42 to 0.05)	0.119
Other	0.31 (-0.03 to 0.64)	0.071	0.21 (0.10 to 0.52)	0.176	0.22 (-0.17 to 0.62)	0.294	0.25 (-0.10 to 0.61)	0.157

Note: Significant values: p < 0.05.

^a^ Yellow or Indigenous

^b^ Specialty center, public polyclinic or health center; public hospital/outpatient clinic.

^c^ Crude regression coefficient.

^d^ Regression coefficient adjusted for sociodemographic variables and health service use with p < 0.20 in bivariate analysis.

^e^ Test for linear trend.

Regarding ultra-processed patterns among children aged six to 11 months, greater adherence was directly associated with children covered by health insurance and inversely associated with Yellow or Indigenous ones from lower-income families with who lived in the Brazilian Northeast and visited private health services. In children aged one year or older, greater adherence to ultra-processed patterns was directly associated with Black or Brown children residing in the Brazilian South and inversely associated with the richest ones who lived in the Northeast and visited private health services. After adjustment, the associations for children aged six to 11 months remained significant, except for lower incomes and health insurance possession. Children older than one year showed an association which lost significance for lower incomes and private health services. However, we found associations with the Brazilian Southeast and Midwest (Tables 4 and 5).

## DISCUSSION

Representative data for Brazilian children under two years of age enabled us to find two food intake patterns, which we classified as minimally processed and ultra-processed. The greater adherence of children between six and 11 months to the minimally processed pattern related to higher family incomes and urban residencies in the more developed regions of Brazil (South, Southeast, and Midwest). In children aged 12 months or older from higher family incomes and living in more developed regions showed higher adherence, an association we also found among those who were white and used private childcare services. Regarding ultra-processed patterns among children aged six to 11 months, greater adherence was inversely associated with Yellow or Indigenous children who lived in the Brazilian Northeast and used private health services. At 12 months or more, greater adherence was directly associated with Black or Brown children who resided in more developed regions, and inversely associated with those living in the Brazilian Northeast.

The minimally processed pattern we found explained the highest proportion of total variance and best represented the food intake of Brazilian children under two years of age. This pattern showed foods or derivatives related to non-breast milk, fruits, meat or eggs, and vegetables in general. Our findings add information to previous findings from some regional studies conducted in Brazil and abroad with children of different ages. These studies also found a type of dietary pattern, consisting mostly of recommended food groups^16,22^. An example was the “healthy pattern” found in children aged 13 to 35 months in a Northeastern capital, which included vegetables, tubers, meat, offal, rice, pasta, fruit, and fruit juices^16^. A study with two- to nine-year-old schoolchildren in the Brazilian Southeast observed another similar food pattern, “traditional food,” which consisted of six groups of consumed foods: meat, grains, beans, milk and dairy products, vegetables, and fruits^22^. Moreover, research with Australian children under the age of two^23^and with European children between two and nine years of age^24^ also found patterns consisting of food groups resembling our minimally processed pattern.

Brazilian children tend toward a healthy eating pattern, aligned with the recommendations of the *Guia Alimentar para Crianças Brasileiras Menores de 2 anos* (Dietary Guidelines for Brazilian Children under 2 Years of Age^1^). These Guidelines recommend that, from six months of age onward parents or guardians should introduce their children to an adequate and healthy complementary diet to breast milk, based on fresh or minimally processed foods directly obtained from plants and animals, such as fruits, vegetables, legumes, eggs, meats, tubers, grains, and cereals^1^. Parents and guardians should also start encouraging and adopting healthy eating practices and preventing chronic diseases in children’s later stages of life from their first two years of age onward since eating practices acquired during childhood tend to continue in adulthood^25^.

Regarding the consumption of non-breast milk or its derivatives, within the minimally processed pattern, we should mention that parents or guardians should avoid supplying children under one year of age with non-breast milk^1^. In special situations, a qualified healthcare provider should guide the supply of non-breast milk^1^. However, as it was impossible to evaluate whether the consumption of this food group followed the recommendations of the Ministry of Health^1^, research should insert the minimally processed dietary pattern in its analyses with caution.

The second pattern we found, “ultra-processed pattern,” included artificial juices, sweets, candies or other sugary foods and soft drinks. Similarly, a study with children aged 13 to 35 months, conducted in Brazilian Northeast urban households, found a dietary pattern its authors called “unhealthy”, which included artificial juices, soft drinks, cookies, simple cakes, and unhealthy snacks^16^. A study conducted with schoolchildren from the Southeast found a pattern its authors called “ultra-processed foods,” consisting of fast food, artificial juice, snacks, sugary snacks, cookies/cakes with filling, and lower vegetable intake^22^. Note that some differences in the composition of dietary patterns between these studies may result from the different instruments available to obtain data on children’s food intake, the evaluated age group, and the region in which authors conducted their studies, which also increases the relevance of performing this study in a nationally representative sample of children under two years of age.

Since we found a general high prevalence of consumption of many ultra-processed foods, we stress the importance these findings may represent in the health of children under two years of age. Our results indicate that more than three quarters of these children had consumed cookies or cake, data higher than that in a previous study^14^. Also, more than half of the children we evaluated consumed sugary drinks, agreeing with results from a study conducted in the Brazilian Southeast^26^. Moreover, we observed that about 40% of Brazilian children under two years of age consumed sweets, candies or other sugary foods; a pattern observed in children before four months of age^14^. The consumption of ultra-processed foods is associated with unfavorable outcomes to childhood health, such as obesity and dyslipidemias^7,8,24^.

As described in the *Guia Alimentar para Crianças Brasileiras Menores de 2 anos* (Dietary Guidelines for Brazilian Children under 2 Years of Age^1^), the ultra-processed dietary pattern we identified is unrecommended for children under two years of age. Although the literature has reported the damage associated with consuming ultra-processed foods^7,24,27^, findings in Brazil and abroad indicate the growing consumption of this food group, especially among children in different geographic regions and socioeconomic scenarios^26,28^. The consumption of ultra-processed foods is associated with children’s poor diet, especially those rich in sugar^29^, sodium^29,30^, and saturated fats^30^ and those low in vitamins^26,30^, fibers, proteins, and potassium^5^.

Furthermore, longitudinal studies evince that preschoolers^7,8,24^ and adolescents^27^ in Brazil and other countries consume ultra-processed foods, a diet associated with increased central adiposity^7,27^, altered lipid profile^8^, and increased levels of C-reactive protein, a biomarker commonly associated with adiposity and cardiovascular risk factors^24^. The early introduction of ultra-processed foods can also impair exclusive breastfeeding since the supply of this type of food begins before children’s fourth month of life^14^. Our results reinforce the need for more effective actions to monitor Brazilian children’s growth and development, especially regarding guidelines aimed at introducing complementary public or private food services.

Analyses performed for both age groups (six to 12 months and 12 to 24 months) found no differences in dietary pattern composition but evinced that Brazilian children already consumed ultra-processed foods as young as one year of age. A result which resembles that from a multicenter study conducted with children living in Southern Brazil^14^, which found that the consumption of non-recommended foods begins even before children’s first year of life.

Regarding the adherence of children under two years of age to the minimally processed pattern and higher household incomes, a study conducted in a Brazilian Northeastern capital found similar results^17^. However, other studies which used comparable methods failed to find the adherence of white children living in the more developed urban areas of Brazil.

We found that non-white children older than one year showed the highest adherence to the ultra-processed pattern, a result resembling a study with North American children in this age group^31^. Regarding the inverse association between the ultra-processed pattern and children living in the Brazilian Northeast, a previous analysis, which evaluated the prevalence of consumption of sugary drinks in children under two years of age via 2013 PNS data^32^found comparable evidence. However, the ultra-processed dietary pattern not only was inversely associated with children living in the Northeast (as observed in a previous study^32^) but was also directly related to children living in more developed regions, suggesting regional differences in the composition of dietary patterns. Unlike a previous finding^14^, we observed no association between the ultra-processed pattern and *per capita* family income.

In general, some health, nutritional, and socioeconomic factors (among others) have made it difficult for children from poor countries to achieve their expected development^33^. Research has especially observed that maternal educational attainment and income can interfere in food quality improvement. Moreover, mothers’ educational attainment can influence children’s adherence to both recommended and unrecommended dietary practices^33^.

We have so far found no publication evaluating children’s eating patterns and health service use in a nationally representative sample. Our results indicate that the greatest adherence to adequate and healthy eating patterns were associated with children who visited private childcare services. This agrees with a regional study conducted in the Brazilian Northeast^17^.

In general, health services, such as childcare, can contribute to several positive outcomes by monitoring children’s growth and development; from promoting and recovering children’s health to preventing diseases in childhood and adulthood^17^. Thus, promoting food and nutritional guidance in childcare services is an essential action, especially considering that its lack damages health and is associated with adherence to inadequate eating patterns during childhood^7,8^.

Previous studies indicate that the integrality of childcare is a process under construction in Brazilian Primary Health Care, especially in the services offered by the Family Health Strategy^34^. Moreover, significant changes in the structure of the service and in professionals’ profile are still needed for an effective Family Health Strategy as a model of care^34,35^.

Among the limitations of this study, some were related to the adults taken as a family reference to answer the questionnaire on food consumption. Since they were randomly selected, they may only be indirectly responsible for the child in some households. Furthermore, the use of only one questionnaire on food consumption in the 24 hours prior to data collection may fail to represent children’s usual consumption. However, the use of a large sample with PNS data may have minimized the effect of food consumption variability. Other limitations regard the use of factor analysis, including the following arbitrary options: number of components to be extracted, rotation method, and component naming^36^. The observed patterns, however, cohere with the main behaviors which promote chronic non-communicable diseases in Brazil and abroad.

Despite its limitations, this study has important strengths. Our results reinforce the current scientific literature since they found two eating behavior patterns in Brazilian children which are associated to opposite recommendations in the *Guia Alimentar para Crianças Brasileiras Menores de 2 anos* (Dietary Guidelines for Brazilian Children under 2 Years of Age^1^). Moreover, we showed that the demographic and economic factors to which children are exposed may influence these patterns. The literature has no national population-based study evaluating outcomes with similar methods (i.e., complex probabilistic sampling and methodological rigor), enabling us to extrapolate our results for the entire infant population under two years of age in Brazil.

Considering our findings, the consumption of some ultra-processed foods showed a high prevalence in Brazilian children under two years of age. We found two dietary patterns of different compositions in this population, which may be distributed according to several sociodemographic characteristics and health service use. Moreover, these patterns seem to represent two opposite recommendations in the *Guia Alimentar para Crianças Brasileiras Menores de 2 anos* (Dietary Guidelines for Brazilian Children under 2 Years of Age) for food intake and items to be avoided. This may guide the implementation of health promotion actions, including those already in force, so children achieve results with greater equity.
