# Supplementary material for: Characteristics associated with dietary patterns in Brazilian children under two years of age
Source: Rev Saude Publica. 2022 Nov 18;56:118. doi: 10.11606/s1518-8787.2022056003757 (PMC9749735; doi:10.11606/s1518-8787.2022056003757)
Supplement: Translation

# Translation: Características associadas a padrões alimentares em crianças brasileiras menores de dois anos

**Authors:** Rumão Batista Nunes de Carvalho, Maria Laura da Costa Louzada, Fernanda Rauber, Renata Bertazzi Levy

**Affiliations:** I Brasil Universidade Federal do Piauí. Faculdade de Enfermagem. Picos, PI, Brasil; II Brasil Universidade de São Paulo. Faculdade de Saúde Pública. Núcleo de Pesquisas Epidemiológicas em Nutrição e Saúde. São Paulo, SP, Brasil; III Brasil Universidade de São Paulo. Faculdade de Medicina. Departamento de Medicina Preventiva. São Paulo, SP, Brasil

**Keywords:** Nutrição do Lactente, Alimentos, Dieta e Nutrição, Fatores Socioeconômicos, Serviços de Saúde da Criança

## Abstract

**OBJETIVO:**

Analisar padrões alimentares de crianças brasileiras menores de dois anos e verificar a sua associação com características sociodemográficas e de utilização de serviços de saúde.

**MÉTODOS:**

Estudo transversal com dados da Pesquisa Nacional de Saúde (PNS) de 2013. A identificação dos padrões foi realizada em dois grupos etários por meio da análise fatorial por componentes principais e a correlação às características de interesse, testada por meio de modelos de regressão linear.

**RESULTADOS:**

Em ambos os grupos foram identificados dois padrões alimentares: o primeiro foi caracterizado pelo consumo de alimentos *in natura* ou minimamente processado e o segundo marcado somente pelo consumo de alimentos ultraprocessados. A maior adesão das crianças entre seis e 11 meses ao primeiro padrão foi associada à maior renda familiar *per capita*, residência em área urbana e nas regiões mais desenvolvidas do país. Com 12 meses ou mais, a adesão foi relacionada com a raça/cor branca, maior renda familiar *per capita*, residência nas regiões mais desenvolvidas e realizar consultas de puericultura em serviços privados. No segundo padrão, a aderência entre os menores de um ano foi inversamente associada com raça/cor amarela ou indígena, residência na região Nordeste e realização de puericultura nos serviços públicos especializados ou nos privados. A partir dos 12 meses, a adesão foi diretamente associada com raça/cor preta ou parda e residência nas regiões mais desenvolvidas, e inversamente associada com residência na região Nordeste.

**CONCLUSÃO:**

O estudo identificou dois padrões alimentares opostos em crianças brasileiras menores de dois anos, sendo que diferentes determinantes sociais modificam a chance de adesão a esses padrões.

## INTRODUÇÃO

Os dois primeiros anos de vida são cruciais para o crescimento e desenvolvimento da criança, e nesta fase a alimentação adequada e saudável desempenha um papel fundamental para que esses processos ocorram de forma satisfatória^1^. Desde que seja ofertada em quantidade e qualidade apropriada, a alimentação associa-se às melhores condições de saúde na infância, assim como pode atuar na prevenção de diversas doenças desenvolvidas na fase adulta^1^.

Por outro lado, a alimentação inadequada é o mais importante fator de risco para anos de vida perdidos e está associada a inúmeras doenças crônicas não transmissíveis altamente prevalentes, como obesidade, diabetes tipo II, hipertensão e alguns tipos de câncer^2^. Além disso, é um dos principais determinantes da sindemia global de obesidade, desnutrição e mudanças climáticas, que afeta a maioria das pessoas em todos os países e regiões do mundo^3^. Essas consequências são particularmente relevantes para crianças, já que comportamentos e agravos de saúde adquiridos em fases iniciais da vida tendem a repercutir em idades posteriores^4^.

Estudos prévios evidenciam que um dos principais determinantes da piora da qualidade da alimentação decorre, principalmente, da substituição do consumo de refeições saudáveis, baseadas em alimentos *in natura* ou minimamente processados, por alimentos prontos ou semiprontos para consumo, identificados como ultraprocessados^5,6^. Na população infantil, o consumo de alimentos não saudáveis além de estar aumentando, parece estar associado ao maior risco de desenvolver obesidade^7^ e dislipidemia^8^, sendo que a primeira é o fator metabólico mais comum no desenvolvimento de outros agravos crônicos entre as crianças, como diabetes e pressão arterial elevada^4,9^.

Esses achados são especialmente importantes, já que nos primeiros anos de vida, além da introdução de alimentos complementares, essencial para atender às necessidades nutricionais e de desenvolvimento de uma criança, também é uma época em que práticas parentais, preferências e hábitos alimentares, saudáveis ou não , poderão ser estabelecidos^10^, o que, ainda, demanda a necessidade da avaliação contínua de marcadores de consumo nessa faixa etária.

Além disso, até o momento são poucos os estudos que avaliam a influência da utilização de serviços de saúde na alimentação infantil. Em geral, as pesquisas que avaliaram mães usuárias desse suporte têm se concentrado em identificar os aspectos relacionados ao monitoramento e desempenho de indicadores, especialmente os de amamentação^11,12^, assim como a existência de vínculo entre as usuárias e o profissional do serviço^13^. Ainda, discute-se a ideia de que a prática de introdução de alimentos não recomendados no primeiro ano de vida se associa à falta de orientações nutricionais eficazes aos responsáveis, por parte dos profissionais de saúde, como também à baixa aderência às ações de prevenção e educação nutricional implementadas, especialmente em serviços de atenção básica^14^. Esse cenário também leva a realizar investigações mais aprofundadas.

Considerando que o consumo alimentar tende a ser mais bem representado pela análise do efeito simultâneo de diferentes grupos de alimentos^15^, estudos anteriores, realizados em escolares ou em residentes de determinadas regiões do país, avaliaram a alimentação por meio da identificação de padrões alimentares e observaram que características maternas tendem a influenciar a adesão a esses padrões^16,17^. No entanto, esses achados não permitem generalizar os padrões alimentares e os fatores sociodemográficos associados a todo o território nacional, e para todas as faixas de idade da população infantil, assim como ainda não foram observadas evidências de padrões alimentares que, nessa fase, podem estar associados à utilização de serviços de saúde. Essa lacuna permite realizar novos estudos que analisem informações em uma amostra nacionalmente representativa de crianças brasileiras, como em menores de dois anos de idade.

Assim, a partir dos dados de amostra populacional relacionados à alimentação infantil, disponibilizados pela Pesquisa Nacional de Saúde em 2013^18^, e dos métodos robustos de análise exploratória, que permitem identificar e explicar as combinações mais frequentes do consumo alimentar, este estudo se propõe a analisar padrões alimentares de crianças brasileiras menores de dois anos e verificar a sua associação com características sociodemográficas e de utilização de serviços de saúde.

## MÉTODOS

### Delineamento, População de Estudo e Coleta de Dados

Estudo transversal, de base populacional domiciliar, que utiliza dados secundários da Pesquisa Nacional de Saúde, conduzida pelo Instituto Brasileiro de Geografia e Estatística (IBGE), em parceria com o Ministério da Saúde. A coleta de dados foi realizada por entrevistadores treinados e ocorreu no período de agosto de 2013 a fevereiro de 2014.

A PNS utilizou amostragem probabilística em três estágios: no primeiro, os setores censitários constituíram as unidades primárias de amostragem; o segundo estágio referiu-se ao sorteio dos domicílios; e o terceiro, ao sorteio de um morador de 18 anos ou mais de idade. Em cada estágio, foi realizado sorteio por amostragem aleatória simples. Os setores censitários ou conjuntos utilizados na PNS foram sorteados com base no Sistema Integrado de Pesquisas Domiciliares do IBGE, usando Amostra Mestra, com maior espalhamento geográfico e ganho de precisão das estimativas. Desta forma, a amostra da PNS é representativa do Brasil, macrorregiões, unidades da federação, regiões metropolitanas, capitais e o restante das unidades da federação.

Foram obtidos registros de entrevistas em 60.202 domicílios (taxa de resposta de 86,1%). As crianças menores de dois anos de idade foram identificadas no segundo estágio, quando um número fixo de domicílios particulares permanentes em cada unidade primária de amostragem foi selecionado^19^. As mães ou responsáveis responderam às questões referentes às crianças nos domicílios sorteados. Neste estudo, apenas as 3.646 crianças entre seis e 23 meses de idade foram incluídas na amostra final.

Definiram-se pesos amostrais para as unidades primárias de amostragem, domicílios e todos os seus moradores. Detalhes adicionais sobre o processo de amostragem, fatores de ponderação, coleta e outras informações podem ser encontrados em publicação anterior^19^.

### Variáveis de Estudo

Dados relacionados ao consumo alimentar, às características sociodemográficas e de utilização de serviços de saúde foram analisados.

#### Consumo Alimentar

O consumo alimentar foi obtido por meio de resposta a um questionário aplicado ao responsável pelo domicílio da criança que avaliou o consumo dos seguintes alimentos nas 24 horas que antecederam o dia da entrevista: leite não materno ou derivados; frutas ou suco natural; verduras/legumes; feijão ou outras leguminosas (lentilha, ervilha etc.); carnes ou ovos; batata e outros tubérculos e raízes (batata doce, mandioca); cereais e derivados (arroz, pão, cereal, macarrão, farinha etc.); sucos artificiais; biscoitos, bolachas ou bolo; doces, balas ou outros alimentos com açúcar; e refrigerantes. Todas essas variáveis foram categorizadas em indicadores dicotômicos (0 = não consumiu; 1 = consumiu).

#### Características Sociodemográficas

Consideraram-se as seguintes variáveis sociodemográficas: sexo (masculino; feminino); idade (seis a 11 meses e 29 dias; 12 a 23 meses e 29 dias); raça/cor da pele referida (branca; preta ou parda; outras – amarela ou indígena); renda familiar *per capita* (distribuída em quintos); área de residência (rural; urbana); e região do país (Norte; Nordeste; Sudeste; Sul; Centro-Oeste).

#### Características de Utilização de Serviços de Saúde

As variáveis de utilização de serviços de saúde incluíam: cobertura de plano de saúde (sim; não); e local de acompanhamento do crescimento e desenvolvimento (puericultura) (não faz acompanhamento; unidade básica de saúde; serviço público especializado – centro de especialidades, policlínica pública ou posto de assistência médica, hospital público/ambulatório; consultório particular ou clínica privada; e outros).

## Análise dos Dados

A distribuição sociodemográfica e de utilização de serviços de saúde e a prevalência dos alimentos consumidos foram apresentadas em percentuais e respectivos intervalos de confiança de 95% (IC95%) para a amostra total.

Os padrões de comportamento alimentar foram definidos utilizando análise fatorial por componentes principais^15^, método indicado quando os dados não apresentam distribuição normal^20^. Em seguida, os fatores obtidos foram rotacionados por meio de rotação ortogonal varimax. Os testes de esfericidade de Bartllet e Kayser-Meyer-Olkin (KMO) foram aplicados para verificar a adequação dos dados para realização da análise fatorial^21^. Os fatores retidos atenderam os critérios de autovalor (*eigenvalue*) > 1 e análise gráfica *scree plot*. A melhor interpretabilidade dos fatores foi utilizada para composição final dos componentes, e os alimentos com cargas fatoriais maiores ou igual a 0,3 foram considerados importantes para a identificação do padrão alimentar. Alimentos que apresentaram cargas cruzadas (*cross-loadings*) – carregamentos de itens maior ou igual aos valores de carga especificados (0,3) em dois ou mais fatores – foram retirados da análise para identificação e discriminação do melhor ajuste aos dados na estrutura fatorial^20^. Em seguida, os escores fatoriais foram preditos para cada criança avaliada.

A associação entre os escores preditos (variável dependente) e as variáveis sociodemográficas e de utilização de serviços de saúde (independentes) foi avaliada mediante modelos de regressão linear. No modelo ajustado, foram incluídas variáveis com p < 0,20 nas análises bivariadas e mantidas apenas as significativas (p < 0,05). O ajuste do modelo foi verificado pela análise de distribuição de resíduos, que apresentou normalidade.

Realizou-se a análise de dados no software Stata versão 14.0, por meio do módulo *survey*, considerando o delineamento amostral. A análise fatorial e a regressão linear foram realizadas na amostra total e na estratificada pela idade em meses.

A PNS foi aprovada na Comissão Nacional de Ética em Pesquisa (Protocolo 328.159, de 26 de junho de 2013). Todos os entrevistados foram consultados e esclarecidos, e aqueles que aceitaram participar da pesquisa assinaram o Termo de Consentimento Livre e Esclarecido.

## RESULTADOS

Foram avaliadas 3.646 crianças brasileiras entre seis e 23 meses (1.249 de seis a 11 meses; 2.397 de 12 a 23 meses). A maioria era do sexo masculino (53,3%), tinha entre 12 e 23 meses (68,3%), foi declarada preta ou parda (51,9%) e pertencia ao primeiro e segundo quintos da distribuição de renda familiar *per capita* (52,6%). Crianças que residiam na área urbana (83,8%), nas regiões Nordeste (29,4%) e Sudeste (37,2%), sem cobertura de plano de saúde (73,6%), atendidas nas unidades básicas de saúde (55,4%) e em serviços privados (27,9%) apresentaram as maiores frequências na distribuição (Tabela 1).

**Tabela 1:** Descrição das características da amostra de crianças de seis meses a menores de dois anos de idade. Pesquisa Nacional de saúde (PNS), 2013 (n = 3.646).

Características	Percentual (%)	IC95%
**Sociodemográficas**		
Sexo		
Masculino	53,3	50,7–55,8
Feminino	46,7	44,2–49,3
Idade		
6–11 meses e 29 dias	31,7	29,3–34,1
12–23 meses e 29 dias	68,3	65,9–70,7
Raça/cor		
Branca	47,1	44,6–49,6
Preta ou parda	51,9	49,3–54,4
Outras^a^	1,0	0,5–1,9
Renda familiar *per capita*		
1º quinto (menor renda)	26,9	25,0–29,0
2º quinto	25,7	23,5–28,0
3º quinto	21,5	19,5–23,6
4º quinto	13,5	11,8–15,5
5º quinto (maior renda)	12,4	10,5–14,4
Área de residência		
Rural	16,2	14,8–17,6
Urbana	83,8	82,4–85,2
Região do País		
Norte	11,0	10,3–11,8
Nordeste	29,4	27,9–31,0
Sudeste	37,2	35,5–38,9
Sul	15,6	14,4–16,9
Centro-Oeste	6,8	6,2–7,3
**Utilização de serviços de saúde**		
Cobertura de plano de saúde		
Sim	26,4	24,0–29,0
Não	73,6	71,0–76,0
Puericultura		
Não faz acompanhamento	7,3	6,1–8,8
Unidade básica de saúde	55,4	52,6–58,0
Serviço público especializado^b^	6,7	5,5–8,1
Consultório particular ou clínica privada	27,9	25,4–30,5
Outros	2,7	2,0–3,7

^a^ Amarela ou indígena

^b^ Centro de especialidades, policlínica pública ou posto de assistência médica; hospital público/ambulatório.

A descrição dos alimentos consumidos pelas crianças está apresentada na Tabela 2. Os maiores percentuais de consumo de alimentos considerados *in natura* ou minimamente processados foram observados para cereais e derivados (83,1%), frutas ou suco natural de frutas (81,5%) e leite não materno ou derivados (80,5%). Entre os alimentos ultraprocessados, o maior percentual de consumo foi de biscoitos/bolachas/bolo (76,6%); mais da metade das crianças menores de dois anos (52,4%) tinha consumido sucos artificiais e refrigerantes nas últimas 24 horas.

**Tabela 2:** Percentual de consumo nas 24 horas anteriores à entrevista de alimentos na amostra de crianças de seis meses a menores de dois anos de idade. Pesquisa Nacional de Saúde (PNS), 2013 (n = 3.646).

Alimentos consumidos	Percentual (%)	IC95%
Cereais e derivados (arroz, pão, cereal, macarrão, farinha etc.)	83,1	81,1–84,9
Frutas ou suco natural	81,5	79,5–83,3
Leite não materno ou derivados	80,5	78,3–82,5
Feijão ou outras leguminosas (lentilha, ervilha etc.)	79,3	77,2–81,2
Biscoitos, bolachas ou bolo	76,6	74,3–78,7
Carnes ou ovos	76,1	73,9–78,2
Verduras/legumes	74,8	72,6–76,9
Batata e outros tubérculos e raízes (batata doce, mandioca)	61,1	58,6–63,5
Doces, balas ou outros alimentos com açúcar	39,5	36,9–42,1
Sucos artificiais	31,7	29,4–34,1
Refrigerantes	20,7	18,7–22,9

No geral, a verificação da adequação dos dados obteve valor de KMO 0,80 e teste de Bartlett com p < 0,001, indicando adequação dos dados para realização da análise fatorial.

Pela análise foi possível identificar dois fatores, tanto em toda a amostra quanto naquela estratificada segundo a faixa etária. Os dois fatores explicaram 46% da variância compartilhada nas crianças entre seis e 11 meses e 41% nas crianças mais velhas. Na Tabela 3 são apresentadas as cargas fatoriais rotacionadas em cada um dos dois componentes, para a amostra total e para a estratificada. O consumo de “biscoitos, bolachas ou bolo” apresentou *cross-loading* e foi retirado da análise para melhor validade de discriminação entre os fatores.

**Tabela 3:** Cargas fatoriais rotacionadas para os dois componentes da análise fatorial por componentes principais, estratificados por idade. Pesquisa Nacional de Saúde (PNS), 2013.

	6–11 meses e 29 dias (n = 1.249)			12–23 meses e 29 dias (n = 2.397)			Total da amostra (n = 3.646)		
		
	Padrão minimamente processado	Padrão ultraprocessado	Porcentagem comum^a^	Padrão minimamente processado	Padrão ultraprocessado	Porcentagem comum	Padrão minimamente processado	Padrão ultraprocessado	Porcentagem comum
**Alimentos consumidos**									
Leite não materno ou derivados	**0,35**	-0,07	0,87	**0,38**	-0,02	0,86	**0,36**	0,04	0,87
Frutas ou suco natural	**0,64**	-0,05	0,59	**0,57**	-0,09	0,67	**0,60**	-0,06	0,63
Verduras/legumes	**0,75**	-0,07	0,44	**0,71**	-0,06	0,49	**0,73**	-0,08	0,46
Feijão ou outras leguminosas	**0,61**	0,23	0,58	**0,60**	0,16	0,61	**0,60**	0,23	0,59
Carnes ou ovos	**0,69**	0,21	0,47	**0,65**	0,22	0,53	**0,65**	0,28	0,50
Batata e outros tubérculos e raízes	**0,75**	0,00	0,44	**0,63**	0,11	0,60	**0,67**	0,02	0,55
Cereais e derivados	**0,61**	0,25	0,56	**0,60**	0,26	0,57	**0,60**	0,29	0,56
Sucos artificiais	0,03	**0,69**	0,53	0,06	**0,64**	0,59	0,04	**0,67**	0,56
Doces, balas ou outros alimentos com açúcar	0,18	**0,70**	0,48	0,13	**0,73**	0,45	0,14	**0,74**	0,44
Refrigerantes	-0,02	**0,75**	0,44	0,02	**0,73**	0,46	0,00	**0,73**	0,46
**Variância proporcional (%)**	29,00	17,00	-	25,00	16,00	-	26,00	18,00	-
**Variância acumulada (%)**	29,00	46,00	-	25,00	41,00	-	26,00	44,00	-

^a^ Comunalidade: proporção da variância de cada variável explicada pelos fatores extraídos.

Os itens indicados em negrito apresentaram carga fatorial maior ou igual a 0,3.

Em ambos os grupos etários e para o total da população estudada, o primeiro componente denominado “padrão minimamente processado”, foi caracterizado pelo consumo de alimentos *in natura* ou minimamente processado (leite não materno ou derivados, frutas ou suco natural, verduras/legumes, feijão ou outras leguminosas, carnes ou ovos, batata e outros tubérculos e raízes, e cereais e derivados). Já o segundo, intitulado “padrão ultraprocessado”, foi caracterizado pelos alimentos ultraprocessados (sucos artificiais, doces, balas ou outros alimentos com açúcar e refrigerantes).

Nos modelos brutos, a aderência ao padrão minimamente processado, nas duas faixas de idade, foi maior entre as crianças brancas, de maior renda, residentes das zonas urbanas e das regiões mais desenvolvidas do país, com planos de saúde e atendidas em serviços de saúde privados. Nos modelos ajustados para as crianças de seis a 11 meses, esse padrão manteve-se significantemente associado com famílias de maior renda, residentes das zonas urbanas e das regiões mais desenvolvidas do país. No modelo para crianças de 12 a 24 meses, a maior aderência ao padrão minimamente processado se associou significativamente com as crianças brancas, com maior renda, residentes das regiões mais desenvolvidas do país, e atendidas em serviços de saúde privados (Tabelas 4 e 5).

**Tabela 4:** Associação bruta e ajustada entre os padrões de consumo alimentar em crianças de seis a 11 meses e 29 dias e características sociodemográficas e de acesso a serviços de saúde. Pesquisa Nacional de Saúde (PNS), 2013 (n = 1.249).

Características	Padrão minimamente processado				Padrão ultraprocessado			
	
	β bruto^c^ (IC95%)	p	β ajustado^d^ (IC95%)	p	β bruto (IC95%)	p	β ajustado (IC95%)	p
**Sociodemográficas**								
Sexo								
Masculino	Ref.		Ref.		Ref.		Ref.	
Feminino	-0,08 (-0,25 a 0,09)	0,361	-		-0,03 (-0,19 a 0,13)	0,718	-	-
Raça/cor								
Branca	Ref.		Ref.		Ref.		Ref.	
Preta ou parda	-0,28 (-0,42 a -0,13)	< 0,001	-0,00 (-0,16 a 0,15)	0,971	0,12 (-0,05 a 0,29)	0,152	0,11 (-0,06 a 0,29)	0,190
Outras^a^	-0,07 (-0,68 a 0,54)	0,821	-0,15 (-0,81 a 0,52)	0,666	-0,52 (-0,89 a -0,14)	0,007	-0,40 (-0,77 a -0,02)	0,039
Renda domiciliar *per capita*								
1º quinto (menor renda)	Ref.		Ref.		Ref.		Ref.	
2º quinto	0,51 (0,29 a 0,74)	0,000^e^	0,31 (0,10 a 0,53)	< 0,001^e^	0,06 (-0,15 a 0,28)	< 0,001^e^	0,07 (-0,15 a 0,29)	0,060^e^
3º quinto	0,76 (0,56 a 0,96)		0,44 (0,21 a 0,68)		-0,02 (-0,30 a 0,26)		0,01 (-0,30 a 0,32)	
4º quinto	0,83 (0,63 a 1,03)		0,43 (0,18 a 0,68)		-0,17 (-0,37 a 0,03)		-0,13 (-0,38 a 0,11)	
5º quinto (maior renda)	1,03 (0,83 a 1,23)		0,53 (0,27 a 0,78)		-0,49 (-0,66 a -0,33)		-0,31 (-0,59 a -0,03)	
Área de residência								
Rural	Ref.		Ref.		Ref.		Ref.	
Urbana	0,51 (0,29 a 0,74)	< 0,001	0,40 (0,21 a 0,60)	< 0,001	-0,11 (-0,34 a 0,11)	0,322	-	-
Região do país								
Norte	Ref.		Ref.		Ref.		Ref.	
Nordeste	0,33 (0,12 a 0,55)	0,003	0,35 (0,14 a 0,55)	0,001	-0,26 (-0,46 a -0,05)	0,013	-0,25 (-0,43 a -0,06)	0,010
Sudeste	0,80 (0,58 a 1,03)	< 0,001	0,54 (0,31 a 0,78)	< 0,001	-0,08 (-0,34 a 0,18)	0,528	0,11 (-0,16 a 0,38)	0,421
Sul	0,81 (0,60 a 1,01)	< 0,001	0,54 (0,33 a 0,74)	< 0,001	0,02 (-0,25 a 0,29)	0,895	0,22 (-0,04 a 0,47)	0,096
Centro-Oeste	0,83 (0,61 a 1,05)	< 0,001	0,60 (0,38 a 0,81)	< 0,001	0,06 (-0,22 a 0,35)	0,671	0,20 (-0,07 a 0,78)	0,146
**Utilização de serviços de saúde**								
Cobertura de plano de saúde								
Sim	Ref.		Ref.		Ref.		Ref.	
Não	-0,54 (-0,69 a -0,39)	< 0,001	-0,11 (-0,28 a 0,06)	0,211	0,23 (0,07 a 0,40)	0,006	-0,02 (-0,22 a 0,18)	0,842
Puericultura								
Não faz acompanhamento	Ref.		Ref.		Ref.		Ref.	
Unidade básica de saúde	0,45 (0,13 a 0,76)	0,005	0,15 (-0,16 a 0,46)	0,345	-0,17 (-0,47 a 0,12)	0,252	-0,17 (-0,47 a 0,13)	0,272
Serviço público especializado^b^	0,29 (-0,12 a 0,71)	0,167	0,04 (-0,33 a 0,42)	0,820	-0,41 (-0,78 a -0,03)	0,032	-0,40 (-0,78 a -0,01)	0,043
Consultório particular/clínica privada	0,96 (0,65 a 1,30)	< 0,001	0,30 (-0,04 a 0,65)	0,086	-0,65 (-0,94 a -0,36)	< 0,001	-0,57 (-0,93 a -0,21)	0,002
Outros	0,09 (-0,39 a 0,57)	0,710	-0,04 (-0,44 a 0,37)	0,864	-0,33 (-0,80 a 0,14)	0,167	-0,32 (-0,77 a 0,13)	0,159

^a^ Amarela ou indígena.

^b^ Centro de especialidades, policlínica pública ou posto de assistência médica; hospital público/ambulatório.

^c^ Coeficiente de regressão bruto.

^d^ Coeficiente de regressão ajustado para variáveis sociodemográficas e de utilização de serviços de saúde com p < 0,20 na análise bivariada.

^e^ Teste de tendência linear.

Nota: valores significantes: p < 0,05.

**Tabela 5:** Associação bruta e ajustada entre os padrões de consumo alimentar em crianças de 12–23 meses e 29 dias e características sociodemográficas e de acesso a serviços de saúde. Pesquisa Nacional de Saúde (PNS), 2013 (n = 2.397).

Características	Padrão minimamente processado				Padrão ultraprocessado			
	
	β bruto^c^ IC95%)	p	β ajustado^d^ (IC95%)	p	β bruto (IC95%)	p	β ajustado (IC95%)	p
**Sociodemográficas**								
Sexo								
Masculino	Ref.		Ref.		Ref.		Ref.	
Feminino	0,07 (-0,04 a 0,18)	0,231	-	-	0,06 (-0,06 a 0,19)	0,312	-	-
Raça/cor								
Branca	Ref.		Ref.		Ref.		Ref.	
Preta ou parda	-0,37 (-0,48 a -0,26)	< 0,001	-0,13 (-0,24 a -0,01)	0,030	0,14 (0,03 a 0,26)	0,015	0,17 (0,05 a 0,29)	0,005
Outras^a^	-0,30 (-0,78 a 0,17)	0,213	-0,09 (-0,52 a 0,33)	0,670	0,35 (-0,09 a 0,79)	0,120	0,26 (-0,14 a 0,66)	0,204
Renda domiciliar *per capita*								
1º quinto (menor renda)	Ref.		Ref.		Ref.		Ref.	
2º quinto	0,33 (0,18 a 0,48)	< 0,001^e^	0,23 (0,07 a 0,39)	< 0,001^e^	0,04 (-0,12 a 0,20)	0,012^e^	-0,04 (-0,20 a 0,13)	0,068^e^
3º quinto	0,60 (0,44 a 0,76)		0,38 (0,21 a 0,55)		0,05 (-0,14 a 0,24)		-0,01 (-0,20 a 0,18)	
4º quinto	0,72 (0,56 a 0,89)		0,45 (0,27 a 0,63)		0,07 (-0,12 a 0,27)		0,03 (-0,19 a 0,25)	
5º quinto (maior renda)	0,90 (0,74 a 1,06)		0,54 (0,34 a 0,74)		-0,37 (-0,57 a -0,18)		-0,38 (-0,64 a -0,13)	
Área de residência								
Rural	Ref.		Ref.		Ref.		Ref.	
Urbana	0,39 (0,25 a 0,53)	< 0,001	0,13 (-0,02 a 0,27)	0,082	0,03 (-0,10 a 0,16)	0,648	-	-
Região do país								
Norte	Ref.		Ref.		Ref.		Ref.	
Nordeste	0,30 (0,11 a 0,49)	0,002	0,30 (0,12 a 0,48)	0,001	-0,17 (-0,32 a -0,02)	0,022	-0,16 (-0,31 a -0,02)	0,027
Sudeste	0,70 (0,50 a 0,89)	< 0,001	0,43 (0,24 a 0,63)	< 0,001	0,08 (-0,09 a 0,26)	0,339	0,25 (0,07 a 0,43)	0,006
Sul	0,77 (0,58 a 0,96)	< 0,001	0,54 (0,35 a 0,73)	< 0,001	0,24 (0,05 a 0,44)	0,016	0,41 (0,20 a 0,62)	< 0,001
Centro-Oeste	0,72 (0,52 a 0,93)	< 0,001	0,53 (0,33 a 0,73)	< 0,001	0,12 (-0,08 a 0,32)	0,254	0,23 (0,03 a 0,43)	0,026
**Utilização de serviços de saúde**								
Cobertura de plano de saúde								
Sim	Ref.		Ref.		Ref.		Ref.	
Não	-0,47 (-0,58 a -0,36)	< 0,001	-0,06 (-0,20 a 0,08)	0,395	0,14 (-0,00 a 0,29)	0,053	0,03 (-0,15 a 0,21)	0,734
Puericultura								
Não faz acompanhamento	Ref.		Ref.		Ref.		Ref.	
Unidade básica de saúde	0,27 (0,06 a 0,48)	0,011	0,16 (-0,03 a 0,35)	0,093	-0,03 (-0,23 a 0,16)	0,738	-0,05 (-0,25 a 0,15)	0,614
Serviço público especializado^b^	0,29 (0,00 a 0,58)	0,047	0,16 (-0,12 a 0,43)	0,261	0,00 (-0,26 a 0,26)	0,985	-0,01 (-0,27 a 0,24)	0,922
Consultório particular/clínica privada	0,76 (0,55 a 0,97)	< 0,001	0,27 (0,05 a 0,49)	0,015	-0,23 (-0,44 a -0,01)	0,043	-0,19 (-0,42 a 0,05)	0,119
Outros	0,31 (-0,03 a 0,64)	0,071	0,21 (0,10 a 0,52)	0,176	0,22 (-0,17 a 0,62)	0,294	0,25 (-0,10 a 0,61)	0,157

^a^ Amarela ou indígena.

^b^ Centro de especialidades, policlínica pública ou posto de assistência médica; hospital público/ambulatório.

^c^ Coeficiente de regressão bruto.

^d^ Coeficiente de regressão ajustado para variáveis sociodemográficas e de utilização de serviços de saúde com p < 0,20 na análise bivariada.

^e^ Teste de tendência linear.

Nota: valores significantes: p < 0,05.

Quanto ao padrão ultraprocessado, entre as crianças de seis a 11 meses, a maior adesão foi diretamente associada às crianças com planos de saúde e inversamente associada às crianças declaradas amarela ou indígena, com menor renda, residentes na região Nordeste e atendidas em serviços de saúde privados. A partir de um ano, a maior aderência ao padrão ultraprocessado foi diretamente associada às crianças de cor preta ou pardas e residentes da região Sul, e inversamente associada às mais ricas, residentes no Nordeste e atendidas em serviços de saúde particulares. Após os ajustes, entre as crianças de seis a 11 meses, as associações se mantiveram significativas, exceto para menor renda e plano de saúde. A partir de um ano, a associação perdeu significância para menor renda e os serviços de saúde privados, mas foram identificadas outras associações com a região Sudeste e Centro-Oeste (Tabelas 4 e 5).

## DISCUSSÃO

Dados representativos de crianças brasileiras menores de dois anos possibilitaram identificar dois padrões de consumo alimentar, classificados como padrão minimamente processado e padrão ultraprocessado. Nas crianças entre seis e 11 meses, a maior aderência ao padrão minimamente processado estava relacionada à maior renda familiar *per capita*, residir em área urbana e pertencer às regiões mais desenvolvidas do país (Sul, Sudeste e Centro-Oeste). A partir dos 12 meses, a maior adesão foi mantida entre as crianças com maior renda familiar *per capita* e que pertenciam às regiões mais desenvolvidas, também observada entre aquelas que eram brancas e realizavam consultas de puericultura em serviços privados. Quanto ao padrão ultraprocessado, na faixa etária de seis a 11 meses, a maior adesão estava inversamente associada às crianças declaradas amarela ou indígena, residentes na região Nordeste e atendidas em serviços de saúde privados. Com 12 meses ou mais, a maior aderência foi diretamente associada às crianças de cor preta ou parda e residência nas regiões mais desenvolvidas, e inversamente associada àquelas residentes da região Nordeste.

O padrão minimamente processado, identificado neste estudo, explicou a maior proporção da variância total e foi o que melhor representou o consumo alimentar da população brasileira infantil menor de dois anos. Nesse padrão, foram identificados os alimentos ou derivados relacionados ao leite não materno, frutas e hortaliças em geral, e carnes ou ovos. Nossos achados acrescentam informações ao que foi, até então, observado em alguns estudos regionais realizados no Brasil e de população em outros países, com diferentes faixas etárias da população infantil. Esses estudos também identificaram um tipo de padrão alimentar, composto, em sua maioria, por grupos de alimentos recomendados^16,22^. Um exemplo, foi o “padrão saudável” encontrado em crianças de 13 a 35 meses de idade em uma capital da região Nordeste, que continha itens como, hortaliças, tubérculos, carnes e vísceras, arroz e macarrão, frutas e sucos de fruta^16^. Outro padrão alimentar semelhante, denominado “comida tradicional”, foi observado em estudo com escolares de dois a nove anos na região Sudeste do país, e consistia em seis grupos de alimentos consumidos: carne, grãos, feijão, leite e laticínios, legumes e frutas^22^. Além disso, pesquisas conduzidas com crianças australianas menores de dois anos^23^e com crianças europeias entre dois e nove anos^24^, também encontraram padrões formados por grupos de alimentos semelhantes ao padrão minimamente processado.

É possível observar que a população infantil do Brasil tende a apresentar um padrão alimentar saudável, alinhado às recomendações do Guia Alimentar para Crianças Brasileiras Menores de Dois Anos^1^. Este mesmo Guia recomenda que, a partir dos seis meses, além do leite materno, a introdução de uma alimentação complementar adequada e saudável deverá ser baseada em alimentos *in natura* ou minimamente processados, obtidos diretamente de plantas e animais, tais como as frutas, legumes, verduras, ovos, carnes, tubérculos, grãos e cereais^1^. O incentivo e a adoção de práticas alimentares saudáveis e a prevenção de doenças crônicas em fases posteriores da vida devem iniciar já nos primeiros dois anos de idade, uma vez que as práticas alimentares adquiridas na infância tendem a se manter na vida adulta^25^.

Quanto ao consumo do leite não materno ou de seus derivados, identificado no padrão alimentar minimamente processado, é importante mencionar que a oferta do leite não materno às crianças menores de um ano não é uma prática recomendada quando a criança estiver sendo amamentada^1^. Em situações especiais, a oferta do leite não materno deverá ser orientada por um profissional de saúde capacitado^1^. No entanto, como não foi possível avaliar se o consumo desse grupo de alimentos seguiu as recomendações do Ministério da Saúde^1^, a intepretação do padrão alimentar minimamente processado deverá ser realizada com cautela.

O segundo padrão identificado neste estudo foi denominado “padrão ultraprocessado”, caracterizado pelos seguintes grupos de alimentos: sucos artificiais, doces, balas ou outros alimentos com açúcar, e refrigerantes. De forma semelhante, o estudo com crianças de 13 a 35 meses, realizado em uma região urbana do Nordeste, encontrou um padrão alimentar que chamou de “não saudável”, caracterizado pelo consumo de sucos artificiais, refrigerantes, biscoitos ou bolos simples e lanches não saudáveis^16^. Estudo realizado com escolares da região Sudeste identificou um padrão denominado “alimentos ultraprocessados”, formado pelo consumo de *fast food*, suco artificial, salgadinhos, lanches açucarados, bolachas/biscoitos/bolos com recheio e legumes (carregados negativamente)^22^. Destaca-se que algumas diferenças observadas na composição dos padrões alimentares entre esses estudos podem decorrer dos diferentes instrumentos disponíveis para obtenção dos dados de consumo alimentar das crianças, da faixa de idade avaliada e da região onde foram realizados os estudos, o que ainda aumenta a relevância da realização deste trabalho em uma amostra nacionalmente representativa de crianças menores de dois anos.

Dadas as altas prevalências observadas no consumo isolado de alimentos ultraprocessados neste estudo, ressalta-se a importância que esses achados podem representar na saúde de crianças menores de dois anos. Nossos resultados apontam que mais de três quartos dessas crianças tinham consumido biscoitos, bolachas ou bolo, dados superiores ao encontrado em estudo anterior^14^. Também, mais da metade das crianças avaliadas aqui consumiu bebidas açucaradas, o que está de acordo com os resultados verificados em estudo realizado na Região Sudeste do país^26^. Além disso, observamos que cerca de 40% da população infantil brasileira menor de dois anos consumiu doces, balas ou outros alimentos com açúcar, sendo que o consumo desses tipos de alimentos vem sendo verificado em crianças antes dos quatro meses de idade^14^. O consumo de alimentos ultraprocessados está associado a desfechos desfavoráveis à saúde infantil, como obesidade e dislipidemias^7,8,24^.

Conforme descrito no Guia Alimentar para Crianças Brasileiras Menores de Dois Anos^1^, o padrão alimentar ultraprocessado que identificamos não é o recomendado para as crianças menores de dois anos. Embora os danos associados ao consumo de alimentos ultraprocessados estejam relatados na literatura^7,24,27^, no Brasil e no resto do mundo, os achados indicam um aumento no consumo desse grupo de alimentos, especialmente entre crianças, em diferentes regiões geográficas e cenários socioeconômicos^26,28^. O consumo de alimentos ultraprocessados está associado à má qualidade da dieta infantil, sobretudo com dietas ricas em açúcar^29^, sódio^29,30^e gordura saturada^30^, e com dietas pobres em vitaminas^26,30^, fibras, proteínas e potássio^5^.

Além disso, evidências encontradas em estudos longitudinais, com verificação no Brasil e em outros países, apontam que o consumo de alimentos ultraprocessados entre pré-escolares^7,8,24,^ e adolescentes^27^ está associado ao aumento da adiposidade central^7,27^, à alteração do perfil lipídico^8^ e aos níveis aumentados de proteína C reativa, biomarcador comumente associado à adiposidade e fatores de risco cardiovasculares^24^. Também, a introdução precoce dos alimentos ultraprocessados pode ainda prejudicar o aleitamento materno exclusivo, pois a oferta desse tipo de alimento começa antes do quarto mês de vida da criança^14^. Os resultados reforçam a necessidade de ações mais eficazes no acompanhamento do crescimento e desenvolvimento das crianças brasileiras, especialmente no que se refere a orientações direcionadas a introdução alimentar complementar realizadas em serviços de assistência à saúde, públicos ou privados.

Em relação às análises realizadas para as duas faixas etárias (seis a 12 meses e 12 a 24 meses) não se observou diferenças na composição dos padrões alimentares, mas evidenciou-se que a população infantil brasileira teve acesso ao consumo de alimentos ultraprocessados já a partir de um ano de idade. Resultado semelhante ao que foi encontrado em um estudo multicêntrico realizado com crianças residentes na Região Sul do Brasil^14^, onde se verificou que o consumo de alimentos não recomendados se inicia antes mesmo do primeiro ano de vida.

Quanto à aderência entre o padrão minimamente processado de crianças menores de dois anos e a maior renda domiciliar observada neste trabalho, observou-se similaridade aos resultados encontrados em um estudo realizado numa capital brasileira da região Nordeste^17^. No entanto, a adesão das crianças brancas, que residiam em área urbana e que pertenciam às regiões mais desenvolvidas do país não foi identificada ou avaliada em outros estudos que utilizaram métodos comparáveis.

O padrão ultraprocessado identificado neste estudo teve a maior aderência das crianças maiores de um ano não brancas, estando semelhante aos resultados de um estudo com crianças norte americanas nessa faixa etária^31^. Quanto à associação inversa entre o padrão ultraprocessado e as crianças residentes da região Nordeste, evidências comparáveis foram observadas em análise anterior que avaliou a prevalência de consumo de bebidas açucaradas em menores de dois anos, utilizando também dados da PNS 2013^32^. No entanto, o padrão alimentar ultraprocessado, além de estar inversamente associado às crianças residentes da região Nordeste, como observado no estudo anterior^32^, também foi diretamente relacionado às crianças residentes em regiões mais desenvolvidas, sinalizando que diferenças regionais na composição dos padrões alimentares podem existir. Diferente de achado anterior^14^, a associação entre o padrão ultraprocessado e a renda *per capita* familiar não foi observada neste trabalho.

De modo geral, sabe-se que alguns fatores, como saúde, nutrição e socioeconômicos, têm dificultado que crianças de países pobres alcancem um desenvolvimento esperado^33^. Particularmente, tem-se observado que o nível de escolaridade materno e a renda podem interferir no processo de melhoria da qualidade alimentar, sendo que o nível de escolaridade materna pode influenciar tanto na adesão às práticas alimentares recomendadas quanto àquelas não recomendas para as crianças^33^.

Até o momento, não encontramos nenhuma publicação que tenha avaliado padrões alimentares de crianças e a utilização de serviços de saúde em uma amostra com representatividade nacional. Nossos resultados apontam que as maiores adesões aos padrões alimentares adequados e saudáveis foram associadas às crianças que realizavam consultas de puericultura em serviços privados. E isso está de acordo com o que foi observado em estudo regional brasileiro realizado no Nordeste^17^.

No geral, o acompanhamento do crescimento e do desenvolvimento da criança pelos serviços de saúde, como na puericultura, pode contribuir para desfechos positivos em diversos aspectos, desde a promoção e a recuperação da saúde infantil, até a prevenção de doenças na infância e na vida adulta^17^. E neste contexto, a promoção da orientação alimentar e nutricional inserida nos serviços de puericultura traduz-se em uma ação essencial, principalmente considerando que os danos à saúde e que são associados à adesão aos padrões alimentares inadequados na infância já foram demostrados^7,8^.

Estudos anteriores apontam que a integralidade da atenção à criança é um processo em construção na atenção primária à saúde brasileira, especialmente nos serviços oferecidos pela Estratégia Saúde da Família^34^. Além disso, ainda são necessárias mudanças significativas na estrutura do serviço e no perfil dos profissionais para a efetividade da Estratégia Saúde da Família como um modelo de atenção^34,35^.

Entre as limitações deste estudo, algumas estão relacionadas ao adulto tomado como referência familiar para responder ao questionário sobre o consumo alimentar. O adulto foi selecionado aleatoriamente, e, para alguns domicílios, esse adulto pode não ser o responsável direto pela criança. Ainda, a utilização de apenas um questionário sobre o consumo alimentar das últimas 24 horas pode não representar o consumo habitual da criança. No entanto, como se trata da utilização de uma grande amostra com dados da PNS, o efeito da variabilidade do consumo alimentar pode ter sido minimizado. Outras limitações são tocantes ao uso da análise fatorial, incluindo as seguintes opções arbitrárias: número de componentes a serem extraído, método de rotação e nomeação dos componentes^36^. Os padrões observados, porém, apresentam coerência aos principais comportamentos para doenças crônicas não transmissíveis identificadas no país e em outras regiões do mundo.

Apesar das limitações, este estudo possui importantes pontos fortes. Nossos achados reforçam a literatura científica atual, pois permitiram identificar dois padrões de comportamento alimentar de crianças brasileiras com recomendações opostas verificadas no Guia Alimentar para Crianças Brasileiras Menores de Dois Anos^1^ e que esses padrões podem sofrer influência de fatores demográficos e econômicos, aos quais as crianças estão expostas. Nenhum estudo de base populacional nacional com amostragem complexa probabilística e rigor metodológico foi publicado avaliando desfechos com métodos semelhantes, tornando nossos resultados extrapoláveis para toda população infantil menor de dois anos do país.

Considerando os achados deste estudo, a prevalência de consumo de alguns alimentos ultraprocessados foi alta para a população infantil brasileira menor de dois anos. Nessa população, foram observados dois padrões alimentares de diferentes composições que podem estar distribuídos segundo diferentes características sociodemográficos e de utilização de alguns serviços de saúde. Além disso, esses dois padrões parecem traduzir recomendações opostas às do Guia Alimentar para Crianças Brasileiras Menores de Dois Anos^1^, um como base da alimentação e o outro com alimentos que deveriam ser evitados. Isso pode orientar a implementação de ações de promoção de saúde, incluindo aquelas já utilizadas na infância, a fim de que alcancem resultados com maior equidade.
